# Are there differences in LDL-C target value attainment in Austrian federal states? Yes!

**DOI:** 10.1007/s10354-013-0219-z

**Published:** 2013-08-27

**Authors:** Max Pichler, Dominik Lautsch, Claudia Adler, Karl Bögl, Heinz Drexel, Bernd Eber, Christiane Fauer, Johannes Föchterle, Bernhard Föger, Karin Gansch, Peter Grafinger, Monika Lechleitner, Bernhard Ludvik, Gerald Maurer, Reinhard Mörz, Bernhard Paulweber, Karl Peter Pfeiffer, Rudolf Prager, Gerhard Stark, Hermann Toplak, Otto Traindl, Raimund Weitgasser

**Affiliations:** 1 Private University of the Principality of Liechtenstein, Dorfstrasse 24, 9495 Triesen, Liechtenstein; 2 Merck Sharp & Dohme, Am Europlatz 2, 1120 Wien, Austria; 3 Paracelsus Medical University, Salzburg, Austria; 4 Vorarlberg Institute for Vascular Investigation and Treatment (VIVIT), Feldkirch, Austria; 5 Academic Teaching Hospital Klinikum Kreuzschwestern Wels, Wels, Austria; 6 Linz, Austria; 7 Landeskrankenhaus Bregenz, Bregenz, Austria; 8 General Hospital Linz, Linz, Austria; 9 Landeskrankenhaus Hochzirl, Anna Dengel-Haus, Zirl, Austria; 10 Department of Internal Medicine III, Medical University of Vienna, Vienna, Austria; 11 Department of Internal Medicine II, Division of Cardiology, Medical University of Vienna, Vienna, Austria; 12 Vienna, Austria; 13 Hospital Hietzing, Vienna, Austria; 14 Joanneum, University of Applied Sciences, Graz, Austria; 15 KLI für Stoffwechselerkrankungen, Vienna, Austria; 17 Krankenhaus der Elisabethinen, Graz, Austria; 18 Medical University of Graz, Graz, Austria; 19 Landesklinikum Weinviertel, Mistelbach, Austria; 20 Abteilung für Innere Medizin, Diakonissen-Krankenhaus Salzburg, Salzburg, Austria

**Keywords:** LDL Cholesterol, Regional differences, Ezetimibe, Statin, Coronary Heart Disease, LDL Cholesterin, Regionale Unterschiede, Ezetimib, Statin, Koronare Herzkrankheit

## Abstract

Low density lipoprotein (LDL-C) levels determine the cardiovascular risk. Previous studies indicated an LDL-C target attainment of around 50 %, but no Austrian wide analysis on results for the federal states was available. We therefore sought to detect potential differences.

Design: Open-label, non-interventional, longitudinal study, registered: www.clinicaltrials.gov NCT 01381679. In all, 746 statin treated patients not at LDL-C goal received intensified therapy for 12 months. The sample was split into nine subgroups, representing the federal states of Austria.

We detected an east-west gradient for baseline LDL-C. Individual target values were achieved by 37.2 % (range: 26.1–57.7 %). After 12 months, LDL-C < 70 mg/l was achieved by 13.5 % (5.9–38.5 %). Univariate ANCOVA retrieved significant differences within the states (Upper Austria and Salzburg, *p* = 0.001 and *p* = 0.0015, respectively). Furthermore, the capacity of intensified lipid lowering therapy applied in practice was as high as −42 % as compared to previous standard therapy (additional LDL-C reduction after switch from baseline therapy in Vorarlberg).

## Abstract (de)

Low Density Lipoprotein Cholesterin (LDL-C) Spiegel determinieren das kardiovaskuläre Risiko. Vorangegangene Studien zeigten eine LDL-C Zielwerterreichung von circa 50 %. Nachdem keine österreichweite geographische Analyse zur Zielwerterreichung verfügbar war, ermittelten wir, ob es Unterschiede zwischen den Österreichischen Bundesländern gibt.

Design: Open-label, nicht-interventionelle, longitudinale Studie, registriert unter www.clinicaltrials.gov NCT01381679. 746 statinbehandelte Patienten, die ihren LDL-C Zielwert nicht erreichten, erhielten intensivierte Therapie. Die Stichprobe wurde in neun, die Österreichischen Bundesländer repräsentierenden, Subgruppen unterteilt.

Ein Ost-West Gradient konnte für LDL-C zu Beginn der Studie festgestellt werden. LDL-C Werte < 70 mg/dl wurden von 13,5 % (5,9–38,5 %) der Patienten in Österreich nach 12 Monaten erreicht. Univariate ANCOVA zeigte einen signifikanten Unterschied zwischen den Bundesländern (Oberösterreich und Salzburg, *p* = 0,001, bzw. *p* = 0,0015). Die Kapazität intensivierter, in der Praxis angewandter, lipidsenkender Therapie betrug −42 % im Vergleich zu vorhergehender Standardtherapie (zusätzliche LDL-C Senkung nach Switch von der Therapie zu Beginn der Studie in Vorarlberg).

## Introduction

Elevated cholesterol levels are a paramount risk factor for the development of atherothrombotic events and eventually cardiovascular death [1, 2]. Reaching low density lipoprotein cholesterol (LDL-C) target values can reduce the incident of such events: According to the data of the CTT meta-analysis, a 1 mmol/L (around 39 mg/dl) reduction in LDL-C levels resulted in a 22 % reduction of cardiovascular events and a 10 % reduction of total mortality [3]. Furthermore, evidence from statin, niacin, and ezetimibe studies supports that achieved LDL-C levels < 80 mg/dl can lead to a regression in atheroma volume [4–6]. Both international and Austrian guidelines recommend LDL-C levels between 70 and 100 mg/dl for patients at high or very high cardiovascular risk [7–9]. The most recent European guidelines recommend a target value of 70 mg/dl for virtually all patients with coronary heart disease or diabetes [8, 9]. Previous cross-sectional studies revealed a target attainment of around 50 % in high-risk patients not only in Austria, but all over the western world [10–15].

We acknowledge the interesting, recently published data on geographical differences within Austria for both obesity and cardiovascular mortality [16, 17]. These differences were associated with gradients in diabetes mellitus and hypertension and explained by psychosocial differences that apply to the highly populated, urban eastern Austrian region versus the rural, mountainous west. Furthermore and according to the authors of those studies, the level of education is lower in eastern Austria than in the rest of the country. In order to see whether such differences also exist concerning hyperlipidemia, baseline LDL-C and target value attainment, we aimed to asses geographical aspects within Austria, especially considering differences between the nine federal states.

## Patients, material and methods

The detailed study design of the Austrian cholesterol screening and treatment II (ACT II) study was published elsewhere [18]. In brief, the present paper refers to a population-based, open-label, non-interventional, and observational longitudinal study. Study centers were office based internists and family care physicians who consecutively included patients at high or very high cardiovascular risk on statin therapy and not reaching LDL-C target levels. Subsequently, they received intensified cholesterol reducing therapy at the discretion of the treating physicians in order to reach individual LDL-C targets. After baseline examination two follow-up visits were performed after 3 and 12 months.

Study period was 12 months. The study was performed between 2009 and 2010.

In order to detect differences in LDL-C target achievement, the sample was split into nine distinct groups representing the federal states Burgenland, Carinthia, Lower Austria, Upper Austria, Salzburg, Styria, Tyrol, Vorarlberg, and Vienna. The patients included represent 0.01 % of each state’s population, Table 1 [19].

**Table 1:** Patient numbers in the study in comparison to the states’ population

	Austria	Burgenland	Carinthia	Lower Austria	Upper Austria	Salzburg	Styria	Tyrol	Vorarlberg	Wien
Public census	8,281,295	280,082	559,698	1,587,651	1,403,762	525,859	1,203,036	696,049	363,952	1,661,206
Inclusion in ACT II	746	16	36	213	88	27	164	69	20	113
Percentage (%)	0.01	0.01	0.01	0.01	0.01	0.01	0.01	0.01	0.01	0.01

The present substudy aims to detect differences in LDL-C target attainment after 12 months of intensified medical therapy in the nine Austrian federal states. Reference for LDL-C targets were the former guidelines on cardiovascular disease prevention, issued by the European Society for Cardiology in 2007 [20] and the National Cholesterol Education Program Adult Treatment Panel III (NCEP ATP III) guidelines [21], which were valid and effective during the study. At the time of examination, LDL-C target value <70 mg/dl was in place only for patients suffering from concomitant coronary heart disease (CHD) and diabetes (DM) or metabolic syndrome, acute coronary syndrome (ACS) or progressive heart disease. Target value <100 mg/dl was in place for patients diagnosed with CHD (only), Type 2 DM, Type 1 DM and nephropathy, extracoronary atherosclerosis, or an ESC SCORE <5 %. As mentioned, the most recent guidelines set LDL-C  <70 mg/dl as the target value for all of these patients, reflecting current evidence from large outcome trials.

Adult, statin treated patients who did not meet LDL-C target values were included in the study. This selection of patients was based on the Austrian reimbursement rules in order to reflect practice conditions in this non-interventional study. Austrian reimbursement rules are defined by the *Erstattungskodex* (*EKO*, local reimbursement compendium) [22]. Exclusion criteria were contraindications to any of the drugs used in the course of the non-interventional study and an age <18 years.

LDL-C was calculated using the Friedewald Formula in standardized Austrian medical labs. Statistical calculations were performed with SPSS 17.0. For comparisons between the states we used ANOVA, for regression analyses univariate models in a stepwise approach (ANCOVA), as further explained in the result section. Values were collected using paper based case report forms, the electronic transfer was performed by the Koordinierungszentrum für Klinische Studien, Medical University Innsbruck. No missing values were added post hoc or calculated by regression to the mean nor supplemented by other methods. Adverse events were documented precisely and coded according to MedDRA Version 13.1.

The study was approved by the institutional review boards and presented to the *Leit-Ethikkomission der Stadt Wien* (ethics commission). The 1964 Declaration of Helsinki was fully respected. Patients gave consent to the documentation of their anonymized data, treating doctors acted as responsible parties. Registration: www.clinicaltrails.gov registration number NCT 01381679.

## Results

The study included patients from all nine Austrian federal states. While 1,682 patients were included in the study, a complete record of the three examinations (baseline, follow-up 1 and follow-up 2) and allocation of the center to a federal state is available for 746 Patients, treated in 389 centers (GP and internists offices). The results focus only on these 746 patients. Distribution over the nine Austrian states was as followed: Burgenland *n* = 16, Carinthia *n* = 36, Lower Austria *n* = 213, Upper Austria *n* = 88, Salzburg *n* = 27, Styria *n* =164, Tyrol *n* = 69, Vorarlberg *n* = 20, Vienna *n* = 113.

At baseline and for Austria, the distribution of the major lipid parameters was as follows: mean LDL-C was 145 ± 35 mg/dl (all values given are means standard deviation, SD), total cholesterol 236 ± 45 mg/dl, HDL-C 50 ± 15 mg/dl, and triglycerides were 198 ± 120 mg/dl. There were no significant differences within the states with the exception of HDL cholesterol where differences were highly significant at *p* = 0.0001, Table 2. With regard to body weight and waist circumference, data ranged from 77 ± 12 kg (Vorarlberg) to 85 ± 14 kg (Carinthia), and 99 ± 12 cm (Vorarlberg) to 102 ± 14 cm (Lower Austria), respectively. Those differences were significant (*p* = 0.001, Table 3).

**Table 2:** Parameters at baseline in nine Austrian federal states

	Total cholesterol (mg/dl)			LDL Cholesterol (mg/dl)			HDL Cholesterol (mg/dl)			Triglycerides (mg/dl)		
	Baseline	FU 1	FU 2	Baseline	FU 1	FU 2	Baseline	FU 1	FU 2	Baseline	FU 1	FU 2
Burgenland (*n* = 16)	262 ± 60	209 ± 53	204 ± 77	163 ± 43	117 ± 37	96 ± 24	67 ± 28	59 ± 25	65 ± 26	147 ± 78	145 ± 62	135 ± 58
Carinthia (*n* = 36)	245 ± 51	197 ± 54	194 ± 42	146 ± 37	102 ± 35	107 ± 26	45 ± 11	48 ± 12	50 ± 13	234 ± 141	193 ± 102	161 ± 56
Lower Austria (*n* = 213)	233 ± 45	188 ± 38	181 ± 35	146 ± 34	104 ± 31	95 ± 28	48 ± 12	50 ± 13	52 ± 13	205 ± 127	164 ± 93	149 ± 73
Upper Austria (*n* = 88)	230 ± 46	186 ± 49	174 ± 36	140 ± 33	97 ± 32	88 ± 26	52 ± 15	56 ± 15	56 ± 15	180 ± 87	168 ± 74	160 ± 80
Salzburg (*n* = 27)	229 ± 44	181 ± 42	172 ± 27	130 ± 25	89 ± 22	84 ± 20	54 ± 13	58 ± 17	57 ± 14	181 ± 78	152 ± 65	159 ± 88
Styria (*n* = 164)	240 ± 46	188 ± 36	179 ± 38	148 ± 34	103 ± 33	97 ± 26	50 ± 14	54 ± 14	54 ± 15	191 ± 112	158 ± 69	153 ± 81
Tyrol (*n* = 69)	231 ± 36	190 ± 29	182 ± 24	140 ± 38	106 ± 25	96 ± 21	50 ± 13	52 ± 12	56 ± 18	188 ± 98	159 ± 55	153 ± 66
Vorarlberg (*n* = 20)	235 ± 40	184 ± 30	176 ± 32	154 ± 37	100 ± 30	90 ± 24	50 ± 18	52 ± 11	57 ± 15	178 ± 61	159 ± 45	150 ± 38
VIenna (*n* = 113)	238 ± 41	194 ± 41	185 ± 36	144 ± 34	107 ± 35	99 ± 27	48 ± 16	51 ± 15	51 ± 16	221 ± 157	173 ± 91	161 ± 74
Austria (*n* = 746)	236 ± 45	189 ± 40	181 ± 37	145 ± 35	103 ± 32	95 ± 26	50 ± 15	53 ± 14	54 ± 15	198 ± 120	164 ± 81	154 ± 74
*Differences between the states*	n.s. (0.141)	n.s. (0.266)	*0.02*	n.s. (0.440)	n.s. (0.07)	*0.005*	*0.0001*	*0.001*	*0.001*	n.s. (0.052)	n.s. (0.363)	n.s. (0.872)

*FU 1* follow-up visit 1, 3 months after baseline, *FU 2* follow-up visit 2, 12 months after baseline, *n.s.* statistically not significant; all values given as mean ± SD

**Table 3:** Parameters at baseline in respect to the nine Austrian federal states

	Body weight (kg)	Waist circumference (cm)	SysBP (mmHg)	DiaBP (mmHg)	Age (years)
Burgenland	84 ± 18	101 ± 14	138 ± 15	84 ± 9	64 ± 9
Carinthia	85 ± 14	101 ± 11	135 ± 13	83 ± 7	60 ± 10
Lower Austria	84 ± 14	102 ± 14	136 ± 15	80 ± 9	64 ± 10
Upper Austria	83 ± 14	96 ± 13	136 ± 14	82 ± 8	64 ± 10
Salzburg	79 ± 16	99 ± 17	135 ± 18	81 ± 10	66 ± 11
Styria	82 ± 14	99 ± 11	135 ± 15	81 ± 9	64 ± 11
Tyrol	79 ± 14	101 ± 12	133 ± 16	81 ± 9	66 ± 10
Vorarlberg	77 ± 12	99 ± 12	136 ± 11	82 ± 9	64 ± 11
Vienna	82 ± 16	101 ± 13	136 ± 15	81 ± 9	62 ± 11
Austria	82 ± 15	100 ± 13	136 ± 15	81 ± 9	64 ± 10
*Differences between the states*	*0.001*	*0.0001*	n.s. (0.457)	n.s. (0.110)	*0.0001*

*SysBP* Sitting systolic blood pressure, *DiaBP* Sitting diastolic blood pressure; all values given as mean ± SD

Mean LDL-C dropped from the baseline value 145 ± 35 mg/dl to 95 ± 26 mg/dl after 12 months therapy. Values ranged from 130 ± 25 mg/dl (Salzburg) to 163 ± 43 mg/dl (Burgenland) at baseline (differences between states not significant; Table 2). At follow up visit 1 LDL-C dropped to 89 ± 22 mg/dl (Salzburg) to 117 ± 37 mg/dl (Burgenland) and at follow-up visit 2 to 84 ± 20 mg/dl (Salzburg) to 107 ± 26 mg/dl (Carinthia). While no significant differences could be detected at baseline and at follow-up visit 1, differences in the second follow-up visit were highly significant at *p* = 0.005 (ANOVA). For further details please refer to Table 2.

In all, 44.6 % of the patients could be classified as patients at highest cardiovascular risk and 55.4 % as patients at high cardiovascular risk (both groups would be considered to be at very high cardiovascular risk in the recent ESC/EAS guidelines on dyslipidemias) Target values were then defined as <70 and <100 mg/dl respectively. While these individual targets were achieved in 37.2 % of the population, we found differences in target attainment within the nine federal states: Data ranged from 26.1 % (Vienna) to 57.7 % (Salzburg). Values above the median of 39.0 % were achieved in Burgenland (53.3 %), Upper Austria (40.9 %), Salzburg (57.7 %), and Vorarlberg (40.0 %),. LDL-C levels <100 mg/dl were reached by 60.8 % of all Austrian patients at follow-up visit 2. There is a high variance between the Austrian federal states, ranging from 38.2 % (Carinthia) to 76.9 % (Salzburg).

**Fig. 1:**
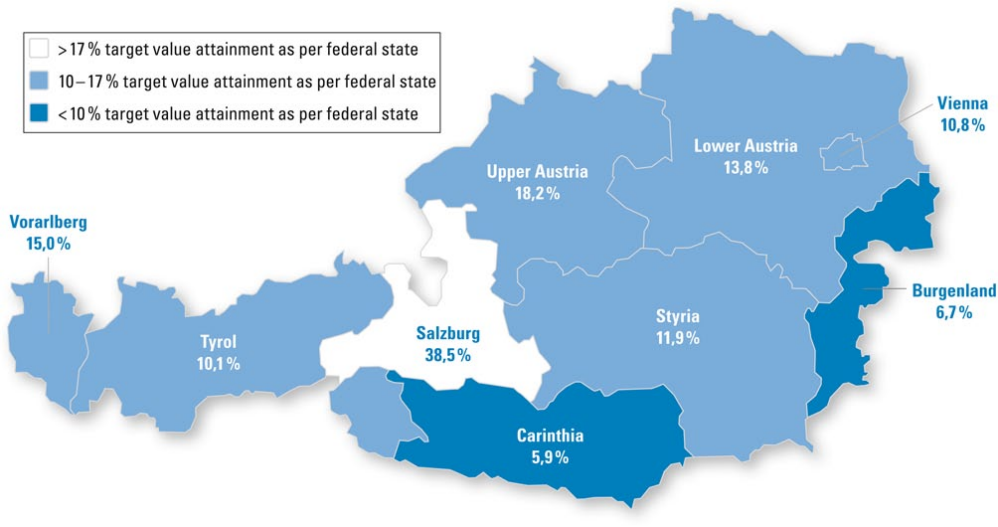
Attainment of low density lipoprotein cholesterol values <70 mg/dl (current target value as set by the major European societies) after 12 months

Most important, LDL-C levels <70 mg/dl (the current standard targets for these groups of patients) were attained by 13.5 % of the Austrian sample with an analogous variance for the states ranging from 5.9 % (Carinthia) to 38.5 % (Salzburg). Details are displayed in Fig. 1.

An analysis of covariance (ANCOVA) was performed in a stepwise approach in order to test the influence of the possibly relevant, examined parameters weight, age, waist circumference, systolic and diastolic blood pressure, as well as body mass index (dependent variable of ANCOVA were the achieved LDL-cholesterol values in follow-up visits 1 and 2). For the baseline LDL-C values, we could retrieve an influence of weight (regression co-efficient beta −0.62, *p* = 0.001), waist circumference (regression co-efficient beta −0.62, *p* = 0.001) and age (regression co-efficient beta −0.62, *p* = 0.001) only. There was no significant influence by the federal states themselves but we observed a positive trend (towards high LDL-C levels) for Burgenland (*p* = 0.051) and a negative trend (towards low LDL-C levels) for Salzburg (*p* = 0.052).

According to these results we then performed a univariate analysis of covariance including the federal states, weight, age, and body circumference only. While results for baseline LDL-C levels did not change in a meaningful way, we could determine an influence of age only at follow-up visit 1 after 3 months (regression co-efficient beta −0.31, *p* = 0.01) but not after 12 months. At this stage, we detected a significant influence of the federal state of Salzburg (*p* = 0.006). After 12 months and considering follow-up visit 2, none of the remaining parameters weight, waist circumference, or age had a statistically significant or trend wise influence on LDL-C levels (*p* = 0.29, *p* = 0.58, and *p* = 0.09). Three federal states remained independently and statistically significant strongly associated with the achieved LDL-C values: Carinthia (regression co-efficient beta 11.43, *p* = 0.024), Upper Austria (regression co-efficient beta −9.88, *p* = 0.01), and Salzburg (regression co-efficient beta −16.25, *p* = 0.004).

To further validate the results we looked at the influence of baseline LDL-C levels on target attainment at follow-up visits 1 and 2. After 3 months the effect was highly relevant and significant (regression co-efficient beta 0.42, *p* < 0.001) but decreased—still significant—to 0.22 (*p* < 0.001) after 12 months. Even after inclusion of this strong parameter, baseline LDL-C, two federal states remained to have the described, very strong influence on LDL-C levels: Upper Austria (regression co-efficient beta − 9.40, *p* = 0.01) and Salzburg (regression co-efficient beta − 13.14, *p* = 0.015), while a positive trend was observed for Carinthia (regression co-efficient beta 8.65, *p* = 0.07).

Lipid therapy at baseline was, as previously reported [18], Simvastatin 62 %, Atorvastatin 10 %, Fluvastatin 8 %, Lovastatin 1 %, Pravastatin 9 %, Rosuvastatin 6 %, and any other therapy at 4 %. During the study, primarily Ezetimibe/Simvastatin fixed dose combinations were used (10/20 mg as most widely use dosage: 45–81 %, least value in Vienna, highest one in Salzburg). Statins in monotherapy (between 0–13 % per state) were administered at intermediate doses. No significant differences in the treatment regimens between the Austrian federal states could be detected.

The documentation of adverse events includes three serious adverse events under fixed dose ezetimibe simvastatin combination therapy. They were not related to the lipid lowering therapy administered. A detailed assessment was published elsewhere [18].

## Discussion

**Fig. 2:**
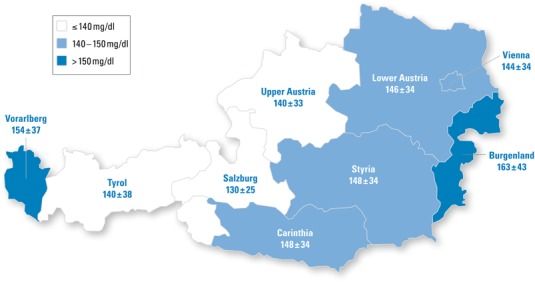
Low density lipoprotein cholesterol levels at baseline of the study (mg/dl), illustrating the described east west gradient in Austria

We report significant differences in attaining the LDL-C target value <70 mg/dl between Austrian states ranging from 5.9–38.5 %. We could further identify an east to west gradient in Austria with highest LDL-C baseline levels in the east and lowest in the west (Fig. 2, exception Vorarlberg). Such a gradient was recently similarly shown for obesity and cardiovascular mortality [16, 17]. Higher LDL-C levels at baseline were documented in Vorarlberg, a region (specifically the Rhine valley) similarly industrialized as the areas surrounding the capital city Vienna.

Therapy in this non-interventional study was intensified lipid lowering treatment at the discretion of the physician. The INCROSS [23] study looked at a population treated with statins and not at goal value and compared Rosuvastatin 10 mg to Ezetimibe/Simvastatin 10/20 mg, the therapy most often used (57 %) in this study. While Rosuvastatin 10 mg achieved a −17 % LDL-C reduction, this value was significantly improved by Ezetimibe/Simvastatin to −28 %. In a very similar approach, a −29 % reduction could be achieved after switching patients from Simvastatin 20 mg or Atorvastatin 10 mg to Ezetimibe/Simvastatin 10/20 mg in the EASEGO study [24]. Several studies [25–27] reported a − 20 to − 27 % LDL-C decrease for Ezetimibe, while LDL-C was lowered by −49 % with Rosuvastatin 20 mg and by −42 % by Atorvastatin in the important trials JUPITER [28] and PROVE-IT TIMI 22 [29]. The Treating to New Targets trial [30], comparing high dose Atorvastatin 80 mg vs. standard dose Atorvastatin 10 mg, retrieved a −21 % reduction for 80 vs 10 mg which is comparable to the results of the VOYAGER meta-analysis that showed a −5 to −6 % LDL-C reduction for each statin titration step [31]. In comparison to these clinical data, our results show what was achieved in practice: mean was −31 % [18], however looking at a federal state level this value was improved up to −42 % (Vorarlberg) and −41 % (Burgenland). At the end of the day, the achieved reductions always depend on three factors: the therapy administered, the willingness of the treating physicians to accept new target levels (< 70 mg/dl for most patients in secondary prevention [8, 9]) and the patients compliance. As for pharmacological lipid therapy, the highest tolerated dose of statin therapy in combination with drugs such as ezetimibe is efficacious and should be used in order to reach LDL-C target levels as according to the guidelines [8].

Looking at achieved absolute LDL-C reductions, data range from − 39 to −67 mg/dl or 1 to 1.5 mmol/l. Applying data from the CTT meta-analysis [3] in practice this would equal to a 22–38 % reduction in cardiovascular events with Carinthia at the lower and Vorarlberg and Burgenland at the higher end (s. Table 2). The CTT analysis was performed with statin trials only, but the recent publication of the SHARP trial [32] indicated, these data should also apply to the statin ezetimibe combination therapy.

Fully applying the LDL-C target of <70 mg/dl target and thus implementing the EAS/ESC 2011 guidelines [8], roughly one-third of the population in Salzburg reached these targets while this value decreased for Carinthia to only 6 % (where only one-third of the population reaches the <100 mg/dl threshold). Interestingly, greatest cardiovascular mortality was reported for Burgenland and Vienna with Tyrol, Carinthia and Vorarlberg as states of lowest cardiovascular mortality risk [17]. Of notice, Vienna was the state with notably low target attainment in the present study.

The observed differences between the federal states are puzzling and hard to understand. Even though numbers of patients are not equal for each state, the same percentage of the broad population was included (0.01 %). We could describe differences in baseline LDL-C which can explain the results to some extent, but Salzburg and Upper Austria were independently associated with improved target value attainment. At this point we can only postulate about different doctor-patient relationship or compliance, but this raises more questions than we could answer from the data provided in this non-interventional study.

Limitations of the study were due to its observational rather than interventional, endpoint-driven nature and due to varying patient numbers in the respective Austrian states. In Burgenland, Carinthia, Salzburg, and Vorarlberg, less than 60 patients were included. These are comparable small numbers, but we included 0.01 % of the populations in all nine Austrian states (Table 1). Yet, the observational nature should be taken as the strength of this study: it reflects real life and thus shows important and interesting differences within the regions of Austria, a small but highly heterogeneous country.

In conclusion, the Austrian analysis of the ACT II study revealed high differences between the nine Austrian federal states in LDL-C levels at baseline and more important, in the achieved target levels. The study furthermore indicates the capacity of intensified lipid lowering therapy versus previous standard therapy applied in practice (up to − 42 %, additional LDL-C reduction after switch from baseline therapy in Vorarlberg).

### Acknowledgements

We would like to acknowledge the work and support of the ACT II study by Philippe Brudi, Sigfried Faber, Christoph Gärtner, Antonia Lang, and Tamara Meixner from Merck, Sharp & Dohme.

### Conflict of interest

B.E. is a consultant and serves on speakers’ bureaus for MSD. B.F. has received speaker fees and/or grant support from Abbott, AstraZeneca, BMS, MSD and Pfizer. K.P. is a statistical consultant for MSD. M.P. has served on speakers’ bureaus and is a consultant for MSD Austria. O.T. is a paid speaker for MSD, AstraZeneca, Takeda and Eli Lilly. RW has received honoraria from MSD as an advisor and lecturer. CA, KB, CF, KG, and DL are employees of MSD. The study was funded by Merck, Sharp & Dohme (MSD).

## References

[1] Anand SS, Islam S, Rosengren A (2008). Risk factors for myocardial infarction in women and men: insights from the INTERHEART study. Eur Heart J.

[2] Castelli WP (1988). Cholesterol and lipids in the risk of coronary artery disease—the Framingham Heart Study. Can J Cardiol.

[3] Cholesterol Treatment Trialists’ Collaboration (2010). Efficacy and safety of more intensive lowering of LDL cholesterol: a meta-analysis of data from 170,000 participants in 26 randomised trials. Lancet.

[4] Nicholls SJ, Ballantyne CM, Barter PJ (2011). Effect of two intensive statin regimens on progression of coronary disease. N Engl J Med.

[5] Bogiatzi C, Spence JD (2012). Ezetimibe and regression of carotid atherosclerosis: importance of measuring plaque burden. Stroke.

[6] Lee JMS, Robson MD, Yu LM (2009). Effects of high-dose modified-release nicotinic acid on atherosclerosis and vascular function. J Am Coll Cardiol.

[7] Austrian Lipid Konsensus. Management of lipid disorders for the prevention of vascular complications. Joint consensus statement of eight Austrian medical societies. 2010.

[8] Task Force for the management of dyslipidaemias of the European Society of Cardiology (ESC) and the European Atherosclerosis Society (EAS). ESC/EAS Guidelines for the management of dyslipidaemias: the Task Force for the management of dyslipidaemias of the European Society of Cardiology (ESC) and the European Atherosclerosis Society (EAS). Atherosclerosis. 2011;217 Suppl 1:S 1–44. 10.1016/j.atherosclerosis.2011.06.012 21723445

[9] European Association for Cardiovascular Prevention, Rehabilitation (EACPR). (2012). European Guidelines on cardiovascular disease prevention in clinical practice (version. The Fifth Joint Task Force of the European Society of Cardiology and Other Societies on Cardiovascular Disease Prevention in Clinical Practice (constituted by representatives of nine societies and by invited experts). Eur Heart J.

[10] Huber K, Roden M (2008). Lipidprofil und Therapiestatus in der Sekundärprävention bei Hochrisiko-Patienten mit klinisch manifester Arteriosklerose und/oder Diabetes mellitus: Das Hospital Screening Projekt (HSP) in Österreich. Wien Klin Wochenschr.

[11] Gitt AK, Drexel H, Feely J (2012). Persistent lipid abnormalities in statin-treated patients and predictors of LDL-cholesterol goal achievement in clinical practice in Europe and Canada. Eur J Prev Cardiol.

[12] Foeger B, Patsch JR (2011). LDL-Cholesterin in der Sekundärprävention: Zielwert-Erreichung unter Lipidsenkern in Praxis und Spital in Österreich (ZIEL). Wien Klin Wochenschr.

[13] Drexel H, Chazelle F, Fauer C, Lautsch D, Gitt AK (2011). Persistent dyslipidemia in Austrian patients treated with statins for primary and secondary prevention of atherosclerotic events—results of the dyslipidemia international study (DYSIS). Wien Klin Wochenschr.

[14] Kotseva K, Wood D, De B (2009). Cardiovascular prevention guidelines in daily practice: a comparison of EUROASPIRE I, II, and III surveys in eight European countries. Lancet.

[15] Waters DD, Brotons C, Chiang CW (2009). Lipid treatment assessment project 2: a multinational survey to evaluate the proportion of patients achieving low-density lipoprotein cholesterol goals. Circulation.

[16] Großschädl F, Stronegger WJ (2012). Regional trends in obesity and overweight among Austrian adults between 1973 and 2007. Wien Klin Wochenschr.

[17] Stein KV, Rieder A, Dorner TE (2011). East-west gradient in cardio-vascular mortality in Austria: how much can we explain by following the pattern of risk factors?. Int J Health Geogr.

[18] Eber B, Lautsch D, Fauer C (2012). Can LDL-cholesterol targets be achieved in a population at high risk? Results of the non interventional study ACT II. Curr Med Res Opin.

[19] Statistik Austria (2011) Bevölkerung sowie Zahl der Gemeinden 2006 nach Gemeindegrößenklassen und Bundesländern. In: Bevölkerung/Volkszählungen, Registerzählung. http://www.statistik.at/web_de/statistiken/bevoelkerung/volkszaehlungen_registerzaehlungen/bevoelkerungsstand/index.html.

[20] Fourth Joint Task Force of the European Society of Cardiology and Other Societies on Cardiovascular Disease Prevention in Clinical Practice (2007). European guidelines on cardiovascular disease prevention in clinical practice: Executive summary. Eur Heart J.

[21] Grundy SM, Cleeman JI, Merz CNB (2004). Implications of recent clinical trials for the National Cholesterol Education Program Adult Treatment Panel III Guidelines. Circulation.

[22] Erstattungskodex (kurz EKO) der Sozialversicherung für die Abgabe von Arzneispezialitäten auf Rechnung eines Sozialversicherungsträgers im niedergelassenen Bereich. http://www.hauptverband.at/portal27/portal/hvbportal/emed/eMedWindow.

[23] Farnier M, Averna M, Missault L (2009). Lipid-altering efficacy of ezetimibe/simvastatin 10/20 mg compared with rosuvastatin 10 mg in high-risk hypercholesterolaemic patients inadequately controlled with prior statin monotherapy—The IN-CROSS study. Int J Clin Pract.

[24] Roeters van Lennep HW, Liem AH, Dunselman PH (2008). The efficacy of statinmonotherapy uptitration versus switching to ezetimibe/simvastatin: results of the EASEGO study. Curr Med Res Opin.

[25] Ben-Yehuda O, Wenger NK, Constance C (2011). The comparative efficacy of ezetimibe added to atorvastatin 10 mg versus uptitration to atorvastatin 40 mg in subgroups of patients aged 65 to 74 years or greater than or equal to 75 years. J Geriatric Cardiol.

[26] Conard SE, Bays HE, Leiter LA (2008). Efficacy and safety of Ezetimibe added on to Atorvastatin (20 mg) versus uptitration of Atorvastatin (to 40 mg) in hypercholesterolemic patients at moderately high risk for coronary heart disease. Am J Cardiol.

[27] Pandor A, Ara RM, Tumur I (2009). Ezetimibe monotherapy for cholesterol lowering in 2,722 people: systematic review and meta-analysis of randomized controlled trials. J Intern Med.

[28] Ridker PM, Danielson E, Fonseca FAH (2008). Rosuvastatin to prevent vascular events in men and women with elevated C-reactive protein. N Engl J Med.

[29] Cannon CP, Braunwald E, McCabe CH (2004). Intensive versus moderate lipid lowering with statins after acute coronary syndromes. N Engl J Med.

[30] LaRosa JC, Grundy SM, Waters DD (2005). Intensive lipid lowering with Atorvastatinin patients with stable coronary disease. N Engl J Med.

[31] Nicholls SJ, Brandrup-Wognsen G, Palmer M, Barter P (2010). Meta-analysis of comparative efficacy of increasing dose of Atorvastatin versus Rosuvastatin versus Simvastatin on lowering levels of atherogenic lipids (from VOYAGER). Am J Cardiol.

[32] Baigent C, Landray MJ, Reith C (2011). The effects of lowering LDL cholesterol with simvastatin plus ezetimibe in patients with chronic kidney disease (Study of Heart and Renal Protection): a randomized placebo-controlled trial. Lancet.

